# Effects of Dietary Protein to Lipid Ratio and Insect Meal on Growth Performance, Feed Utilization, and the Gut Microbiome of Lake Whitefish (*Coregonus clupeaformis*)

**DOI:** 10.1155/anu/5511161

**Published:** 2025-01-24

**Authors:** Rebecca Lawson, Yubing Chen, Junyu Zhang, Marcia A. Chiasson, Jennifer Ellis, Dominique Bureau, Richard D. Moccia, David Huyben

**Affiliations:** ^1^Department of Animal Biosciences, University of Guelph, Guelph, Ontario, Canada; ^2^Life Sciences Centre, Dalhousie University, Halifax, Nova Scotia, Canada; ^3^Ontario Aquaculture Research Centre, University of Guelph, Elora, Ontario, Canada

**Keywords:** 16S sequencing, bacteria, black soldier fly, Canada, Great Lakes, nutrition, rainbow trout, salmonids

## Abstract

Wild stocks of lake whitefish (*Coregonus clupeaformis*) are declining in the Great Lakes, and there is a lack of information on their nutritional requirements and gut health indicators to effectively culture them in an aquaculture setting. The aim of this study was to evaluate the growth performance, nutrient utilization, and gut microbiome of lake whitefish fed varying protein:lipid ratios with and without the inclusion of insect meal from black soldier fly (BSF). In total, 450 lake whitefish (301 ± 10 g) were fed one of five diets with differing protein-to-lipid ratios (high-protein 54%, low-protein 48%, high-lipid 18%, or low-lipid 12%), and an additional commercial control rainbow trout diet (Bluewater commercial control [BCC]). High-protein diets included 5% BSF meal to explore its potential to partially replace fishmeal in the diet. After 16 weeks at 8.5°C, growth performance and nutrient digestibility were the highest for lake whitefish fed the high-protein–high-lipid (HPHL) and BCC diets, while the feed conversion ratio (FCR) was numerically lowest for the HPHL. Protein and energy retention, and lipid digestibility were highest for fish fed the HPHL and BCC diets, while the BCC diet had the highest lipid retained, concomitant with high viscerosomatic index (VSI). High lipid in fish, especially in the viscera that is removed during processing, is not desirable, thus the HPHL diet is recommended. The gut microbiome was dominated by Proteobacteria, specifically by the genera of *Shewanella* and *Aeromonas*, although feeding high-lipid diets resulted in the lowest alpha diversity, but was not significant. These results are novel for this species, and we recommend that lake whitefish diets should be formulated to have a minimum 54:18 protein-to-lipid ratio. The results from this study provide baseline information on the nutrition and gut microbiome of lake whitefish, which can be used to develop a species-specific feed rather than feeding them rainbow trout feed. However, further work on targeted breeding and genetic selection of broodstock, together with diet optimization, is needed to improve the growth performance and nutrient utilization in order to enable an effective, economical, and environmentally sustainable culture of lake whitefish.

## 1. Introduction

Lake whitefish (*Coregonus clupeaformis*) has historically been a valuable species for commercial and Indigenous fisheries in the Great Lakes [1, 2]. However, population abundances and total catches of lake whitefish in the Great Lakes have decreased considerably over the past two decades [3, 4]. In recent years, lake whitefish has become a new species of interest for farming in Ontario, although only one small net-pen facility and a few government culture stations produce lake whitefish in the province [5]. Farming this species for human consumption will augment the commercial fishery industry and may help wild populations to rebound naturally [1]. Farming lake whitefish would also provide consumers with a local, year-round, fresh supply of what is normally a seasonal product [5].

Currently, there are several challenges inhibiting the successful farming of lake whitefish in Ontario and across the Great Lakes. One challenge is that there is presently no commercial lake whitefish feed on the market; thereby, they are commonly fed a commercial diet for rainbow trout since they are a closely related salmonid species [5]. There are no established recommended nutritional guidelines for lake whitefish [6], and the few studies on lake whitefish that do exist focus only on larval feeding [7, 8]. Second, there is presently little information on the gut health of lake whitefish, such as the microbiome, which is important to assess the production of vitamins and fatty acids, metabolic pathways, pathogen transmission, and gut health indicators of this fish species [9]. The few studies that exist that used next-generation sequencing (NGS) to identify the microbiome only used wild and not cultured lake whitefish [10, 11]. Third, if lake whitefish require higher dietary protein levels than rainbow trout, then alternatives to fishmeal, such as insect meal, need to be explored in order to develop sustainable diets for lake whitefish. Replacing fishmeal with black soldier flies (*Hermetia illucens*) has been found to improve the growth performance and the gut microbial diversity of rainbow trout (*Oncorhynchus mykiss*) and Atlantic salmon (*Salmo salar*) [12–14]. More information on the growth performance and gut microbiome of lake whitefish will help optimize production efficiencies and help enable a culture of this species.

The aim of this study was to establish the growth performance, nutrient utilization, and changes in the gut microbiome of lake whitefish fed varying levels of protein and lipid with and without insect meal. We hypothesized that feeding lake whitefish a diet with a high-protein-to-low-lipid (HPLL) ratio can improve the growth performance and feed utilization since they appear to have a large protein intake in the wild (indicative from consuming high amounts of invertebrates). In addition, we hypothesized that chitin from dietary insect meal can increase the diversity of gut microbes by providing pre- and probiotic effects. Prebiotics refer to indigestible fiber that leads to the proliferation of beneficial microbes, while probiotics refer to live microbes in the gut that benefit the host [9]. Therefore, the objective of the current study was to perform a 16-week feeding trial with varying protein:lipid ratios with added insect meal and utilize fish weight measurements, feed intake, proximate analysis, and NGS analysis of the 16S rDNA to investigate growth performance, feed utilization, and gut microbiome of juvenile lake whitefish.

## 2. Materials and Methods

### 2.1. Fish and Facilities

**Figure 1:**
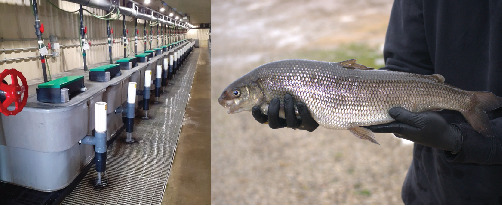
Images of the 15-tank setup and juvenile lake whitefish.

All procedures involving the handling and treatment of fish used in this study were approved by the Animal Care Committee at the University of Guelph under Animal Utilization Protocol #4586. This trial was performed in parallel with another study that focused on the hepatic gene expression and immune response of lake whitefish [15]. More info can also be found in the graduate thesis by Lawson [16]. We conducted the feeding trial at the Ontario Aquaculture Research Centre (OARC) (Elora, ON, Canada), according to Huyben et al. [17]. Lake whitefish were raised from fertilized eggs collected from wild fish from Georgian Bay (ON, Canada). A total of 450 mixed-sex lake whitefish (301 ± 10 g) were randomly allocated into 151-m tanks (330 L), 30 fish/tank, each with a starting density of 27 kg/m^3^ (Figure 1). Fish were acclimated to the tanks and fed a high-quality commercial rainbow trout feed (Bluewater 48-18 MTS, Sharpe Farm Supplies, Guelph, ON, Canada) for 14 days before the start of the experiment. At the start of the trial, the fish were sedated with 70 mg/L tricaine methane sulfonate (Syncaine, Syndel, Nanaimo, BC, Canada), individually weighed, and measured for fork length. Water was sourced from a freshwater well and measured weekly for temperature (8.5°C), dissolved oxygen (10.36 g/L), and total suspended solids (0.1 mg/L). The flow rate was maintained at 10–15 L/min and each tank was equipped with an air stone and shade. The light : dark cycle was 12 h : 12 h, provided by LED lights with a 60 min ramp up and ramp down time to simulate sunrise and sunset.

### 2.2. Diet Formulation and Feeding

Four experimental diets (Table 1) with varying protein : lipid ratios were formulated to meet the nutrient requirements for rainbow trout according to the NRC [6]. The experimental design followed a 2 × 2 factorial (plus commercial control) with high (54%) or low (48%) protein level and high (18%) or low (12%) lipid level. Protein and lipid levels of 48% and 18%, respectively, were used since these levels are used in the commercial rainbow trout diet, and we investigated 54% and 12% protein and lipid levels since we hypothesized the natural diet of wild lake whitefish may consist of higher protein and lower lipid (e.g., insects), which would be more optimal for lake whitefish. In addition, the two high-protein diets contained 5% full-fat black soldier fly (BSF) larvae (Oreka Solutions, Cambridge, ON, Canada) to act as an alternative to fishmeal to feed more sustainable diets, and higher inclusion levels were not used to make these diets more affordable. A commercial control (Bluewater commercial control [BCC]) diet for rainbow trout (Bluewater 48 : 18 MTS, Sharpe Farm Supplies, Guelph, ON, Canada) was used since this pre-existing rainbow trout diet is commonly used to feed lake whitefish since a species-specific diet for lake whitefish did not exist. The BCC control feed was ground and mixed with yttrium oxide (a digestibility marker) and wheat gluten (to help with repelleting) and then repelleted. Feed ingredients were ground to <1 mm. First, dry feed ingredients (Table 1) were mixed for 20 min by an industrial stand mixer. Wet ingredients were added and mixed for an additional 20 min. The mash was heated to 65°C, steam pelleted (California Pellet Mill Co., IN, USA) using a 3 mm die, dried overnight at 40°C, and sieved to remove fines. Diets were stored at 4°C until the start of the trial.

Over 16 weeks (112 days), triplicate tanks of lake whitefish were randomly assigned and fed one of five diets: (1) low-protein–high-lipid (LPHL), (2) low-protein–low-lipid (LPLL), (3) high-protein–high-lipid (HPHL), (4) HPLL, or (5) BCC diet for rainbow trout with LPHL ratio. The LPHL diet was used as an experimental control and formulated to have the same protein:lipid ratio as the BCC diet. Fish were hand-fed to satiety twice a day, 2 days a week, starting at 9:00 and 14:00. For the remaining 5 days, fish were fed using automatic belt feeders twice a day. The automatic feeders were filled with the average amount of feed consumed per day from the two previous hand-feeding days for each individual tank.

### 2.3. Sample Collection

Fish were sedated with 75 mg/L tricaine Syncaine, and individual fish weights and fork lengths were collected at the beginning and the end of the trial and then averaged per tank (*n* = 3 per diet). Bulk weights and feces were stripped from all fish monthly, and feces were stored at −20°C until processing. Six fish from the source population at the beginning of the trial and six fish per tank at the end of the trial were euthanized by an overdose of 400 mg/L Syncaine, followed by severing of the cervical vertebrae. Three carcasses from each tank were pooled per tank and later analyzed for proximate composition (*n* = 3 per diet). Feces from the distal intestine were collected aseptically and squeezed into sterile tubes from two fish per tank (*n* = 6 per diet), as well as a pooled feed sample for each test diet for later microbiome analysis. Total viscera, liver, and visceral fat were weighed from two fish per tank (*n* = 6 per diet) to calculate body indices.

### 2.4. Nutritional Analyses

The feces, diets, and carcass samples were analyzed for proximate composition at the Animal Biosciences Department, University of Guelph (Guelph, ON, Canada) according to AOAC Standard Methods [18]. Feces and carcass samples were ground and then freeze-dried before analysis. Nitrogen was measured using a nitrogen analyzer (FP828, LECO Corporation, St. Joseph, MI, USA), and crude protein was calculated as N × 6.25. Crude lipid was measured using a fat extractor (XT15, ANKOM Technology, Macedon, NY, USA) following the manufacturer's instructions. Gross energy was measured using a bomb calorimeter (C6000, IKA, Wilmington, NC, USA). Moisture was measured by oven drying at 105°C for 16 h, and ash was measured using a muffle furnace at 550°C for 10 h.

Yttrium (digestibility marker) was analyzed by the Lab Services Division, University of Guelph (Guelph, ON, Canada) using nitric acid digestion in closed vessels followed by dilution and measurement of yttrium (321.029 nm) by Inductively Coupled Plasma-Optical Emission Spectrometry (ICP-OES; Agilent 5110, Santa Clara, CA, USA).

### 2.5. DNA Extraction, Amplification, and Sequencing of the Gut Microbiome

DNA extraction of 200–220 mg of feces and feed was performed using QIAamp Stool Mini Kit (Qiagen, Toronto, ON, Canada) according to manufacturer's instructions with additional steps of 15 min heating at 95°C to lyse Gram-positive bacteria and 10 min bead beating with a Tissuelyzer II (Qiagen, Toronto, ON, Canada) for five cycles of 1 min (30 Hz) with 1 min rest in between. DNA was eluted in 10 mM Tris, and the DNA quantity was determined using a Qubit Fluorometer 4.0 (Qubit Systems, Kingston, ON, Canada).

DNA extracts were sent to the Integrated Microbiome Resource (IMR) Lab, Dalhousie University (Halifax, NS, Canada) for library preparation and sequencing of the V3–V4 region of the 16S rRNA gene. In brief, DNA was diluted 1:10 and amplified using Phusion Plus polymerase with nucleotides and buffer (Fisher Scientific, Hampton, NH, USA) with primers 341F (CCTACGGGNGGCWGCAG) and 805R (GACTACHVGGGTATCTAATCC) [19]. A single round of PCR was done using “fusion primers” (Illumina adaptors + indices + specific regions) with multiplexing. PCR products were verified visually by running on a high-throughput Nimbus Select robot using Coastal Genomics Analytical Gels (Hamilton Company, Franklin, MA, USA). The PCR reactions from the same samples were pooled into one plate, then cleaned-up and normalized using the high-throughput Just-a-Plate 96-well Normalization Kit (Charm Biotech, San Diego, CA, USA). Samples were then pooled to make one library, which was then quantified by Qubit before sequencing on the Illumina MiSeq platform using 300 + 300 bp paired-end V3 chemistry (Illumina, San Diego, CA, USA).

The raw 16S rRNA gene sequence reads were deposited in the Sequence Read Archive of NCBI (https://www.ncbi.nlm.nih.gov/sra/PRJNA1119520) and made publicly available under BioProject Accession number PRJNA1119520.

### 2.6. Bioinformatic Analysis of the Gut Microbiome

The 16S rRNA gene sequences (fastq files) were analyzed using Mothur version 1.47.0 [20] according to the MiSeq SOP (https://www.mothur.org/wiki/MiSeq_SOP) [21]. Sequence reads that were larger than 500 bp, had more than eight consecutive bp, and were outside the V3–V4 region of the 16S rRNA were removed from the dataset. Filtered sequence reads were aligned to the SILVA reference database version 138 [22], preclustered to merge sequences with less than 2 bp difference, and chimeras were removed using the open-source tool VSEARCH [23]. Sequences were classified into operational taxonomic units (OTUs) using the SILVA align reference and taxonomy files at a cutoff of 80%, and taxon resembling chloroplasts, mitochondria, and unknowns were removed. Sequences were subsampled to normalize all samples to the lowest number of sequences per sample (i.e., 260). Seven samples with less than 260 sequences were removed, which resulted in sample sizes of 3–6 per diet (i.e., BCC = 6, LPHL = 6, LPLL = 5, HPHL = 3, and HPLL = 3).

### 2.7. Data Analyses of Growth Performance and Nutrition

All values were calculated on a wet matter (WM) basis and averaged per tank according to Bureau et al. [24]:

Nutrient intake (g/fish WM) = mean feed intake × (nutrient%/100),

Weight gain (g/fish WM) = mean final body weight (g (WM)/fish) − mean initial body weight (g (WM)/fish),

Nutrient gain (g/fish WM) = nutrient in final carcass (g (WM)/fish) − nutrient in initial carcass (g (WM)/fish),

Feed conversion ratio (FCR) = mean feed intake (g (WM)/fish)/mean weight gain (g (WM)/fish),

Thermal growth coefficient (TGC) = (final body weight (WM)^1/3^ − initial body weight (WM)^1/3^)/(temperature × day) × 100,

Viscerosomatic index (VSI) = viscera weight (g (WM))/body weight (g (WM)) × 100,

Hepatosomatic index (HSI) = liver weight (g (WM))/body weight (g (WM)) × 100,

Liposomatic index (LSI) = visceral fat weight (g (WM))/body weight (g (WM)) ×100,

Apparent digestibility coefficient (ADC) of dry matter (DM) = [1 − (% yttrium in diet/% *Y* in feces)] 100,

ADC of nutrients (%) = [1− (% *Y* in diet/*Y* in feces) × (% nutrient in feces/nutrient in diet) × 100],

Nutrient retention efficiency (%) = [(final carcass nutrient × final body weight (g (WM))) − (initial carcass nutrient × initial body weight (g (WM)))/(nutrient intake)] × 100.

### 2.8. Statistical Analyses

Statistical analyses were performed using R and RStudio software (version 4.0.3) [25]. Normal distribution and homogeneity of residuals were determined using Shapiro–Wilk and Levene tests. If nonnormal, data were normalized by log transformation. The significance of diet (BCC, LPHL, LPLL, HPHL, and HPLL) was determined using a one-way analysis of variance (ANOVA) (aov function in car package) and the effects of lipid level (high and low) and protein level (high and low) were determined using a two-way ANOVA. The significance of pairwise diet comparisons was estimated using Tukey's adjustment for multiple pairwise comparisons (lsmeans package). Nonnormal data were analyzed using the Kruskal–Wallis test (Kruskal.test function in stats package), and significant effects were estimated using the Dunn test and Benjamini–Hochberg correction for multiple pairwise comparisons (dunn.test package). The gut microbiome alpha-diversity was analyzed using the above ANOVA and Kruskal–Wallis tests based on the Number of Taxa, Shannon (species evenness), and Chao-1 (species richness) indices (vegan package) [26]. Beta-diversity was analyzed using permutation multivariate ANOVA (PERMANOVA) and nonmetric multidimensional scaling (NMDS; vegan package) using the Bray–Curtis index. *p*-Values below 0.05 were considered significant.

## 3. Results

### 3.1. Growth Performance

**Figure 2:**
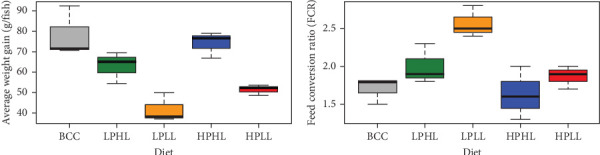
(a) Weight gain and (b) FCR (mean ± SE) of lake whitefish fed diets: Bluewater commercial control (BCC), low-protein–high-lipid (LPHL), low-protein–low-lipid (LPLL), high-protein–high-lipid (HPHL), or high-protein–low-lipid (HPLL) for 16 weeks (*n* = 3 per diet). See Table 2 for significant differences.

The diet had the most significant effects on growth performance, which also resulted in the largest variation between diets (Table 2 and Figure 2). Diet type had significant effects on final fish weight (*p*=0.021), weight gain (*p*=0.001), TGC (*p*=0.007), FCR (*p*=0.006), and VSI (*p*=0.039). Dietary lipid level had significant effects on final fish weight (*p*=0.003), weight gain (*p*=0.001), TGC (*p*=0.002), and FCR (*p*=0.002). Dietary protein level had significant effects on FCR (*p*=0.033), but no other parameter.

Diet type was found to have significant effects on weight gain and FCR (*p*=0.001 and *p*=0.006; Table 2 and Figure 2). Fish fed the BCC and HPHL diets had the largest weight gain (78.2 and 74.1 g/fish) and the lowest FCR (1.70 and 1.63). Fish fed the LPLL diets had the lowest average weight gain (41.7 g/fish) and highest FCR (2.56).

### 3.2. Carcass Composition and Nutrient Utilization

**Figure 3:**
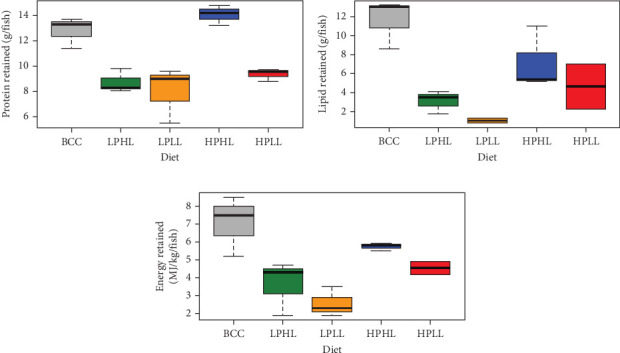
(a) Protein, (b) lipid, and (c) gross energy retention (mean ± SE) in lake whitefish fed diets Bluewater commercial control (BCC), low-protein–high-lipid (LPHL), low-protein–low-lipid (LPLL), high-protein–high-lipid (HPHL), or high-protein–low-lipid (HPLL) for 16 weeks (*n* = 3 per diet). See Table 3 for significant differences.

Diet had many effects on carcass composition and nutrient utilization, especially nutrients retained (Table 3 and Figure 3). Diet type did not have significant effects on the percentage of crude protein, crude lipid, or gross energy in the carcass (*p*=0.095, *p*=0.080, *p*=0.446; Table 3). Fish fed the HPHL diet had the highest percentage of crude protein in the carcass (17.6%), while fish fed the LPHL diet had the lowest percentage (16.7%). Fish fed the BCC diet had the highest percentage of crude lipid and gross energy in the carcass, followed by the HPHL diet, while fish fed the LPLL diet had the lowest percentage.

Diet type was found to have a significant effect on the ADC of DM and lipids (*p*=0.001 and *p*=0.006; Table 3). Dietary protein and lipid levels were found to have a significant interaction effect on lipid ADC (*p*=0.008). Fish-fed high-lipid diets had higher lipid ADC than fish-fed low-lipid diets.

Diet type did not have a significant effect on protein, lipid, or energy intake (*p*=0.230, *p*=0.224, *p*=0.221; Table 3). Fish fed the HPHL diet had the highest protein intake (68.0 g/fish) and energy intake (26 MJ/kg/fish), while fish fed the HPLL diet had the lowest protein intake (44.8 g/fish) and energy intake (14.8 MJ/kg/fish). Lake whitefish-fed high-lipid diets had higher lipid intake than fish-fed low-lipid diets, independent of dietary protein level.

Diet type was found to have a significant effect on crude protein, lipid, and gross energy retention (*p*=0.001, *p*=0.003, *p*=0.008; Table 3 and Figure 3). Dietary lipid level was also found to have a significant effect on protein, lipid, and energy retention (*p*=0.001, *p*=0.02, *p*=0.048). Dietary protein level was found to have a significant effect on protein retention (*p*=0.03), but not lipid or energy retention (*p*=0.215, *p*=0.322). Between treatment diets, the HPHL diet produced the highest amount of protein, lipid, and gross energy retained in the carcass. The LPLL diet retained the least amount of these three components.

### 3.3. Diversity and Composition of the Gut Microbiome

The entire 16S microbiome dataset contained 250,858 sequence reads for 31 samples that included 24 feces, five diets, and two negative blanks (i.e., 8092 sequences/sample). The dataset included 3.3% chimeras and 1.3% nonbacteria (chloroplasts, mitochondria, and unknown sequences), which were removed, resulting in 235,563 sequences. In total, 13 phyla and 245 genera were identified.

Diet type had a significant effect on good's coverage (%) and the number of OTUs in the feed and feces (*p*=0.001, *p*=0.038; Table 4). Fish fed the HPHL diet had the lowest Good's coverage (68.9%), while fish fed the LPHL diet had the highest Good's coverage (96.7%). In addition, fish fed the LPHL diet had the lowest number of OTUs at 13 genera. The HPLL diet had the highest number of OTUs in the intestinal feces at 53 genera. In general, fish had more OTUs in the intestine on Day 112 compared to Day 0, except for the LPHL diet.

**Figure 4:**
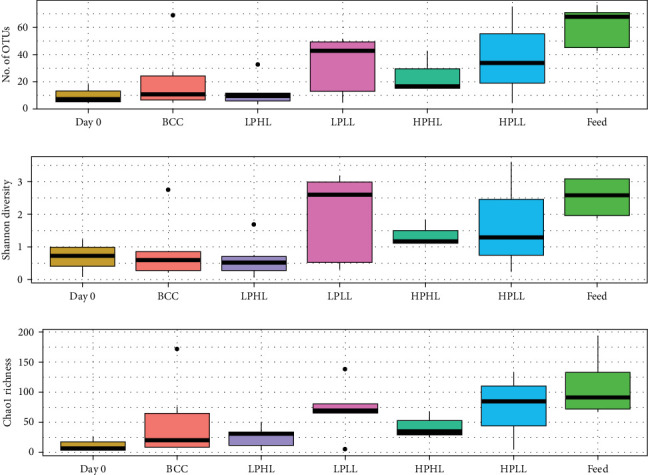
The number of operational taxonomic units (OTUs), Shannon evenness, and Chao1 richness (mean ± SE) of the distal intestinal feces from lake whitefish (Day 0), and after being fed diets Bluewater commercial control (BCC), low-protein–high-lipid (LPHL), low-protein–low-lipid (LPLL), high-protein–high-lipid (HPHL), or high-protein–low-lipid (HPLL) for 16 weeks, and pooled feed (*n* = 3–6 per treatment). See Table 4 for significant differences.

Diet type did not have a significant effect on species richness as measured by the Chao-1 index (*p*=0.088; Table 4 and Figure 4) or on species evenness as measured by the Shannon index (*p*=0.119) in the intestine and feed. Fish fed the HPLL diet had the highest Shannon evenness and Chao-1 richness (1.91 and 151), while fish fed the LPHL diet had the lowest Shannon evenness and Chao-1 richness (0.59 and 34).

**Figure 5:**
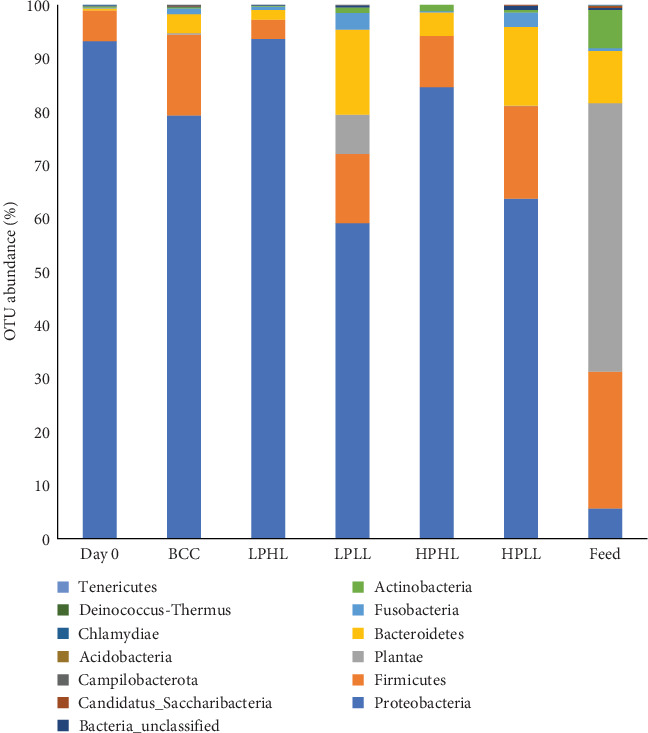
Operational taxonomic unit (OTU) mean relative abundance (on phyla level) of the distal intestine from lake whitefish (*n* = 3–6 per treatment) fed diets Bluewater commercial control (BCC), low-protein–high-lipid (LPHL), low-protein–low-lipid (LPLL), high-protein–high-lipid (HPHL), or high-protein–low-lipid (HPLL) for 16 weeks.

At the start of the experiment (Day 0), the distal intestinal feces were dominated by *Proteobacteria* (93.2%) and *Firmicutes* (5.8%; Figure 5). The top three most abundant phyla in the distal intestinal feces of lake whitefish fed all experimental diets were *Proteobacteria* (77.7%), *Firmicutes* (10.2%), and *Bacteroidetes* (6.3%), respectively, followed by *Fusobacteria* (1.5%) and *Actinobacteria* (0.4%). The most abundant phyla in the feed were *Plantae* (50.3%), *Firmicutes* (25.6%), and *Bacteroidetes* (9.8%). In general, fish-fed low-lipid diets had a higher abundance of *Bacteroidetes* and *Fusobacteria* compared to high-lipid diets (Figure 5). In addition, high-lipid diets had a higher abundance of *Proteobacteria* compared to low-lipid diets.

**Figure 6:**
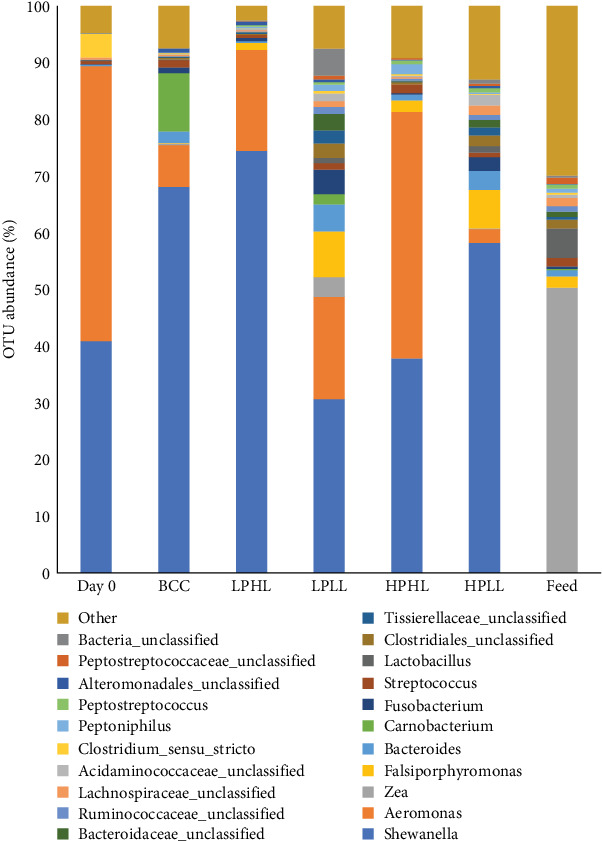
Operational taxonomic unit (OTU) mean relative abundance (on genus level) of the distal intestine from lake whitefish (*n* = 3–6 per treatment) fed diets Bluewater commercial control (BCC), low-protein–high-lipid (LPHL), low-protein–low-lipid (LPLL), high-protein–high-lipid (HPHL), or high-protein–low-lipid (HPLL) for 16 weeks.

**Figure 7:**
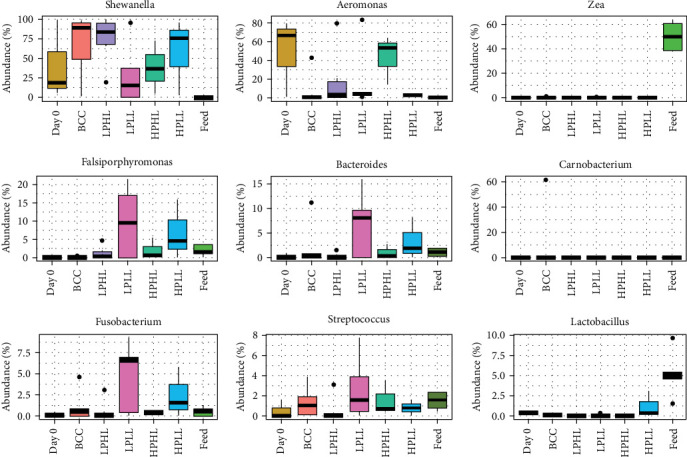
Top 9 of the operational taxonomic unit (OTU) mean relative abundance (on genus level) of the distal intestine from lake whitefish (*n* = 3–6 per treatment) fed diets Bluewater commercial control (BCC), low-protein–high-lipid (LPHL), low-protein–low-lipid (LPLL), high protein high lipid (HPHL), or high-protein–low-lipid (HPLL) for 16 weeks. Shewanella, Zea, and Lactobacillus were significantly different (*p* < 0.05) between feed and all other treatments, while no differences were found for the other bacteria (*p* > 0.05).

On the genus level, the three OTUs with the highest abundance in the distal intestinal feces of lake whitefish fed all diets were *Shewanella* (54.4%) and *Aeromonas* (20.4%; Figures 6 and 7) were not found in the feed. Before the start of the experiment (Day 0), the intestinal feces were dominated by *Aeromonas* (48.5%) and *Shewanella* (40.9%). Fish fed the LPHL diet had the highest abundance of *Shewanella* (74.4%), and the LPLL diet had the lowest abundance of *Shewanella* (30.7%) among experimental diets (Figures 6 and 7). Fish fed the HPHL diet had the largest abundance of *Aeromonas* (43.5%) in the distal intestine, and fish fed the HPLL diet had the lowest (2.4%). *Shewanella*, *Zea*, and *Lactobacillus* were significantly different (*p*  < 0.05) between feed and all other treatments, while no differences were found for the other bacteria (*p*  > 0.05). The following genera were also identified at lower abundances in the feces: *Falsiporphyromonas* (3.2%, *Bacteroides* (2.4%), *Carnobacterium* (2.4%), *Fusobacterium* (1.6%), *Streptococcus* (1.3%), *Clostridium* (0.7%), and *Pseudomonas* (0.1%).

**Figure 8:**
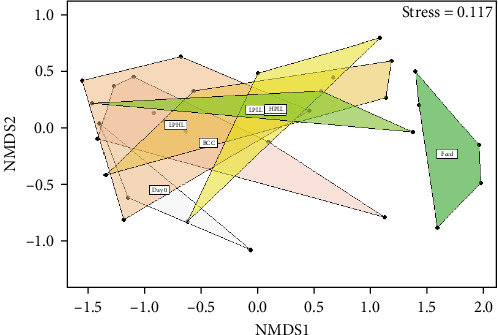
Nonmetric dimensional scaling (NMDS) plots of bacterial beta-diversity from the distal intestine from lake whitefish (*n* = 3–6 per treatment) fed diets Bluewater commercial control (BCC), low-protein–high-lipid (LPHL), low-protein–low-lipid (LPLL), high-protein–high-lipid (HPHL), or high-protein–low-lipid (HPLL) for 16 weeks.

The bacterial community of the feed was different from that in the distal intestinal feces of lake whitefish, shown by the separation of these two materials on the NMDS plot (Figure 8). The NMDS plot of all the diets overlap, representing similarities in the bacterial community of the feces in the distal intestine. Apart from the feed, the Day 0 feces had the least overlap with the feces from fish fed the experimental diets.

## 4. Discussion

This study has provided novel information on the growth performance, feed utilization, and gut microbiome of lake whitefish. The findings from this study can be used to formulate a commercial feed specific for lake whitefish. These results will act as a benchmark and stepping stone for future research to improve the development of lake whitefish as an aquaculture species in Ontario and across the Great Lakes.

### 4.1. Growth Performance Was Highest for BCC and HPHL Diets

Lake whitefish fed the BCC and HPHL diets had the highest growth performance, nutrient digestibility, and nutrient retention. These results do not agree with the first hypothesis that feeding a diet with an HPLL ratio can improve the growth performance, nutrient digestibility, and retention of lake whitefish. However, this is in agreement with a parallel study that found that feeding the HPHL diet to lake whitefish significantly downregulated expression of HSP70 and numerically upregulated expression of catalase [15], which have positive effects on cellular repair and antioxidation [27]. In addition, Chen et al. [15] found that feeding low-lipid diets to lake whitefish increased plasma phosphorus, aspartate aminotransferase, and potassium, which are indicators of poor growth and stress in fish [28, 29]. Lipid is a source of energy and is more energy-dense than proteins since protein metabolism and transport is more energetically demanding, also referred to as the “protein-sparing effect” [17, 30]. Therefore, high-lipid diets may be more energetically optimal for the growth performance of lake whitefish even as the diets were equal in energy (isoenergetic), whereas low-lipid diets result in poor growth and health.

Results from this study are difficult to compare to other studies due to the lack of research on lake whitefish; thus, results were compared to studies focused on European whitefish, which is in the same *Coregonus* genus, as well as rainbow trout and Atlantic salmon, which are in the same Salmonidae family. The highest mean weight gain was only 78 g (HPHL diet), which was very low in comparison to mean weight gains of 174–360 g found in previous studies on European whitefish [31–33], rainbow trout [17], and Atlantic salmon [34] at similar durations and water temperatures. Therefore, the low water temperature of 8.5°C in the present study might only partially explain the low growth. In addition, the fish used in this study were spawned using gametes collected from the wild. This may have been the cause of the low growth rates and high intratreatment variation in growth seen in this study. Potential effects of protein:lipid ratios on growth at higher growth rates may not have been seen in this study. Therefore, future research should be conducted using higher temperatures over a longer experimental period.

Despite the low increase in weight gain, high-lipid (18%) diets produced the best growth performance in lake whitefish, which is similar to previous studies on other salmonid fishes. For example, Ruohonen et al. [31] found that diets with 54% protein, 26% lipids, and 10% carbohydrates resulted in the highest growth rate of European whitefish compared to diets with lower and higher levels of protein and lipids, although diets were not isoenergetic. Gélineau et al. [35] found that diets with 43% protein and 20% lipid resulted in the highest final body weight of rainbow trout without increasing VSI, compared to diets with 15% lipid. Huyben et al. [34] found that diets with 48% protein and 23% lipid along with 1.4% n-3 LC-PUFA resulted in the highest weight gain of Atlantic salmon compared to diets with 18% lipid, although VSI was increased. Aside from studies on other salmonid species, this is the first study to demonstrate a formulated diet with 54% protein and 18% lipid resulted in the highest weight gain for lake whitefish.

In aquaculture, optimizing FCR is necessary for improving the nutritional utilization, economic efficiency, environmental impact, and resource sustainability of feeds [36, 37]. In this trial, FCR ranged from 1.63 (HPHL) to 2.56 (LPLL; Table 2 and Figure 2). Due to genetic and nutrition improvements over the past half-century, juvenile-farmed rainbow trout now have an FCR of around 1.05 depending on their age and rearing conditions [37, 38]. Similarly, farmed Atlantic salmon have an average FCR of 1.20 [36]. The FCR of lake whitefish from this study was nearly double that of current farmed salmonids, demonstrating the importance of the nutritional content of feeds and making a species-specific diet for lake whitefish. In addition, genetic selection for faster growth and more efficient feed utilization through a breeding program will also improve the FCR of lake whitefish.

Low VSI and LSI in fish are indicative of greater somatic growth. Less viscera and lipids around the organs in the abdomen are more ideal for farmers since viscera is removed during degutting and fish fillet processing; thus, visceral weight does not contribute to the degutted fish weight that is priced and sold. The HPHL diet had a lower mean VSI and LSI than the BCC diet (Table 2). In addition, there was a significant interaction between protein and lipid factors where high-lipid diets increased protein and energy retained in the carcass (Table 3 and Figure 3), which suggests that when lake whitefish are provided with high dietary protein levels, larger amounts of protein and lipid are allocated to muscle development rather than energy storage deposited in the viscera. One theory is that lake whitefish can metabolize greater amounts of lipids when fed both high levels of protein and lipid, providing greater amounts of energy for somatic growth since lipid is more energy-dense than protein [39]. In contrast, rainbow trout and Atlantic salmon fed high-lipid diets have resulted in higher VSI and lipid content in the carcass [17, 33, 35, 40], which was not the case in the present study and indicates the lipid level could possibly be higher without jeopardizing growth performance. The lower VSI of fish fed the HPHL diet compared to the BCC diet indicates diets with a higher ratio of protein:lipid than commercial rainbow trout diets, like the BCC diet, produce better growth performance in lake whitefish.

The high-protein diets contained low inclusion of BSF meal at 5% of the total diet, which was not found to reduce growth performance in this study. However, this comparison was difficult to make since the commercial control diet (BCC) was produced using different methods (e.g., extrusion) and had a slightly different formulation than the experimental diets. A meta-analysis was performed on 17 peer-reviewed studies and found that BSF meal can successfully replace 100% of fishmeal (or up to 29% of the diet) without hindering the growth performance of rainbow trout and Atlantic salmon [13, 14]. This finding agrees with our study since our dietary inclusion level of BSF meal was only 5%. Inclusion levels of BSF above 29% of the diet have been found to reduce growth rates of rainbow trout and Atlantic salmon, possibly due to reduced feed intake and protein digestibility [13, 14]. In addition, a meta-analysis by Chen, Ellis, and Huyben [41] of 20 studies found that insect inclusion of 40% was required to significantly stimulate the innate immune response of fish, which also depends on the insect type, tissue analyzed, dietary protein content and fish taxonomic family. Lastly, dietary inclusions of 10%–20% BSF should be investigated for lake whitefish, although the high cost of insect meals compared to fishmeal and plant proteins limits its potential scalability to have higher inclusion of BSF in commercial fish feeds. Therefore, further research on the effects of feeding graded levels of dietary BSF meal on the growth performance and health of lake whitefish is essential.

### 4.2. Nutrient Digestibility and Retention Was Highest for BCC and HPHL Diets

Lake whitefish fed the HPHL diet had significantly higher ADC for DM and lipid compared to the low-lipid diets (Table 3), which indicates the important role dietary lipid level plays in the nutrient utilization of lake whitefish. In contrast, a study on Atlantic salmon found that protein : lipid ratios affected the protein and energy ADC rather than the lipid ADC [34]. Low-protein diets have been reported to cause higher protein ADC in salmonids, such as found in this study. A study on Atlantic salmon fed lower protein:lipid ratios reported the same trend, and the authors contributed this to metabolizing more protein when less lipid is present in the diet [34]. In contrast, a positive correlation between dietary protein level and ADC of protein has been found in Atlantic salmon-fed diets differing in protein-to-lipid ratios as well as replacing fish oil with rapeseed oil [39], although these fish were adults and dietary lipid content was much higher and varied between 35% and 38%. In addition, the high-protein diets also contained a low inclusion of BSF meal, which has been found to reduce the ADC of protein in rainbow trout [14]. However, this trend was not observed in the results from the present study since protein ADC was numerically lower in fish-fed diets, including BSF (high-protein diets) but was not significant (Table 3). It is possible this trend is not present at low inclusions of BSF (5%), whereas significant effects on protein ADC may be found at BSF inclusion of 10% and higher.

The HPHL and BCC diets produced the highest nutrient and energy retention. All five diets were formulated to be isoenergetic, and the HPHL diet was formulated to have the same lipid level. Therefore, the observed difference in lipid and energy retention between lake whitefish fed the BCC and HPHL diets could also be due to differences in manufacturing or the inclusion of different ingredients. The BCC diet was extruded and repelleted and previous studies have found differences in manufacturing can change the digestibility of protein and amino acids in diets for Atlantic salmon [42].

### 4.3. Minor Effects of Diets on Diversity and Composition of the Gut Microbiome

Findings from this study are novel since the gut microbiome of lake whitefish has not been investigated using NGS. The results from this study did not support the hypothesis that increased alpha-diversity in the gut increases the growth of lake whitefish. In addition, low-lipid diets produced a higher alpha-diversity than high-lipid diets, although not significant (Table 4 and Figure 4). A similar result has been reported in Atlantic salmon fed different ratios of protein:lipid and levels of n-3 LC-PUFA, where the lowest alpha-diversity was found in the gut of the fastest-growing fish, while the opposite was true for the highest alpha-diversity [34]. One theory is the high-lipid saturates facultative aerobic bacteria and either inactivates them or reduces their ability to bind to surfaces, referred to as a “soap effect,” leading to reduced diversity in fish fed the high-lipid diets (HPHL and LPHL). Bacteroidetes and Firmicutes decreased in fish fed the high-lipid diets while Proteobacteria increased, which may be due to their tolerance to high concentrations of lipid since they are Gram-negative and have an outer lipid layer while Firmicutes and Bacteroidetes are Gram-positive and have a thick peptidoglycan layer and no outer lipid membrane [43]. Alternatively, the high-protein in the diet acts as a source of nitrogen for bacteria, especially without inactivation from high-lipid, leading to increased diversity in high-protein diets (HPLL and HPHL). This theory is in line with the results where the LPHL diet that had the lowest alpha-diversity.

The inclusion of insect meal in feeds has been found to increase the alpha-diversity (Shannon, Chao-1, and Simpson) of the gut microbiome in rainbow trout [44, 45], which agrees with the results of this current study since the high-protein diets that included BSF resulted in the highest alpha-diversity (Table 4 and Figure 4). Therefore, the results agreed with our hypothesis that feeding chitin, a novel aminopolysaccharide polymer from the exoskeleton of insects, acts as a source of food (substrate) for chitinase-producing bacteria that results in elevated alpha-diversity in the gut microbiome. However, the exact mechanism of chitin increasing the evenness and richness of bacteria in the gut was not verified, and more research is needed. In addition, the low quantity of DNA extracted from lake whitefish feces resulted in the removal of several samples and, consequently, a low sample size. Future studies with optimized DNA extraction and investigation of other host and environmental factors, such as water temperature, would strengthen gut microbiome research on lake whitefish.

Phylum *Proteobacteria* have also been found to dominate the gut microbiome of wild lake whitefish in freshwater lakes in Quebec, Canada. [11], as well as farmed salmonids, such as rainbow trout [12, 46] and Atlantic salmon [47, 48]. Findings from Sevellec, Derome, and Bernatchez [11] show that beta-diversity of the gut microbiome in wild lake whitefish differed significantly between size groups, suggesting the gut microbiome is linked to growth performance in lake whitefish. Interestingly, Sevellec, Derome, and Bernatchez [11] found the smaller lake whitefish had a higher abundance of *Bacteroidetes* in the intestine than normal lake whitefish. This trend is similar to the findings from the current study where lake whitefish fed the LPLL and HPLL diets had the lowest growth performance with the highest abundance of *Bacteroidetes*. In comparison, lake whitefish fed the BCC and HPHL diets had the highest growth performance with the lowest abundance of *Bacteroidetes* (Figure 5). The administration of prebiotic and probiotic supplements has been found to enhance the immune function of the host and increase its resistance to pathogens, thereby enhancing general health and indirectly favoring the feeding and growth of fish [9, 49]. Therefore, abundance of *Bacteroidetes* in the gut microbiome may be an indicator of growth performance in lake whitefish.

The genera identified in the current study have been reported in wild lake whitefish and rainbow trout. Genus *Shewanella* has been reported to dominate wild lake whitefish [11] and rainbow trout [17, 44, 50, 51]. Similar to the current study (Figures 6 and 7), Sevellec, Derome, and Bernatchez [11] found genera Shewanella and *Aeromonas* dominated the gut of wild lake whitefish. However, Sevellec, Derome, and Bernatchez [11] also reported high abundances of *Clostridium* and *Pseudomonas*, which were not identified in the gut of lake whitefish from this study. However, these genera have been found in the gut microbiome of rainbow trout. In addition, genera identified in the current study have also been reported in rainbow trout, such as *Bacteroides*, *Carnobacterium*, and *Streptococcus* [17, 44, 50, 51].

*Shewanella* are a Gram-negative bacteria associated with aquatic environments at low temperatures and the surface of fish [52]. *Aeromonas* are a pathogenic group of bacteria that have also been found in the gut of rainbow trout [51]. *Aeromonas* has also been reported in high abundance in the gut and kidney of wild lake whitefish, associated with disease [10, 11]. Antibiotic treatment and vaccines are difficult since *Aeromonas* strains are known for their enhanced capacity to acquire and exchange antibiotic resistance genes in aquatic environments [53]. A study by Huyben et al. [17] recently found that feeding a blend of organic acids and essential oils was able to reduce the abundance of *Aeromonas* in the gut of rainbow trout. *Falsiporphyromonas* has not been found in lake whitefish before, although its corresponding family, Porphyromonadaceae, has been found in the gut of rainbow trout [17], and *Porphyromonas* has been found in the gut of Arctic charr [54]. Lastly, the feed had a high alpha-diversity and abundance of *Zea*, *Streptococcus*, and *Lactobacillus*, which have been found previously [12].

## 5. Conclusions

Lake whitefish fed the HPHL diet with a protein:lipid ratio of 54:18 exhibited the highest growth performance, protein retention, and lipid digestibility compared to all the other experimental diets; thus, we recommend this diet for future research and culture. Lake whitefish fed the LPLL diet had the lowest growth and nutrient utilization; thus, it is not recommended for lake whitefish culture. These results suggest lake whitefish require a diet formulated with higher protein levels and possibly higher lipid levels than rainbow trout, and we recommend a protein:lipid ratio higher than 54:18 should be investigated. In addition, these results revealed that cultured lake whitefish have a similar gut microbiome to wild lake whitefish, rainbow trout, and Atlantic salmon. Lake whitefish fed high-lipid diets had reduced (non-significant) alpha-diversity compared to low-lipid diets, while the beta-diversity was dominated by genera *Shewanella* and *Aeromonas* with no significant effects of diet. These novel results can be used to formulate a commercial feed for lake whitefish and provide benchmark data on the growth performance of cultured lake whitefish. In addition, this study is the first to investigate the growth performance, feed utilization, and gut microbiome of juvenile lake whitefish in a culture setting and provides critical information for future research and development of this newly cultured species in Ontario and across the Great Lakes. Further research on nutritional requirements, influences on the gut microbiome, and broodstock development is still needed before profitably culturing this species in Ontario.

## Figures and Tables

**Table 1:** Feed formulation and proximate analysis of experimental diets for lake whitefish (*Coregonous clupeformis*).

	Bluewater commercial control	Low-protein–high-lipid	Low-protein–low-lipid	High-protein–high-lipid	High-protein–low-lipid
	BCC	LPHL	LPLL	HPHL	HPLL
Ingredient (% wet matter)					
Fish meal, herring	—	20	20	20	20
Poultry byproduct meal	—	25	25	25	25
Corn gluten	—	10	10	10	10
Wheat flour	—	10	10	10	10
Wheat gluten	5	5	5	5	5
Bluewater feed 48–18	95	0	0	0	0
Blood meal	—	5	5	5	5
Soy protein, HP300	—	8	8	10.5	10.5
Black soldier fly meal	—	0	0	5	5
Corn starch	—	6	11.6	0	5.6
Canola oil	—	5	2.2	3.5	0.7
Fish oil, herring	—	5	2.2	5	2.2
Vitamin and mineral premix	—	0.5	0.5	0.5	0.5
Calcium carbonate	—	0.4	0.4	0.4	0.4
Sea salt	—	0.1	0.1	0.1	0.1
Yttrium oxide	0.015	0.015	0.015	0.015	0.015
Proximate analysis (% dry matter)					
Crude protein	54.6	51.9	51.0	55.8	56.3
Crude lipid	12.9	14.3	9.0	14.6	8.8
Ash	1.0	1.2	1.2	0.9	1.1
Moisture	6.7	6.0	5.9	4.6	5.4
Carbohydrate*⁣*^*∗*^	24.8	26.6	32.9	24.1	28.4
Gross energy (MJ/kg)	21.3	21.7	20.1	21.7	20.3

*⁣* ^*∗*^Calculated as nitrogen free extract (NFE) = 100 − moisture − protein − lipid − ash − fibre.

**Table 2:** Growth performance and body indices of lake whitefish fed the diets, Bluewater commercial control (BCC), low-protein–high-lipid (LPHL), low-protein–low-lipid (LPLL), high-protein–high-lipid (HPHL), or high-protein–low-lipid (HPLL) for 16 weeks.

Growth performance and body indices	BCC	LPHL	LPLL	HPHL	HPLL	SE	*p*-Values		
							Diet	Protein	Lipid
Initial weight (g)	295.2	302.1	303.1	301.6	302.0	10.1	0.914	0.816	0.640
Final weight (g)	373.4^a^	365.0^a,b^	344.8^b^	375.7^a^	353.4^a,b^	15.1	**0.021**	0.175	**0.003**
Fork length (cm)	30.1	30.5	29.9	30.4	30.2	0.4	0.401	0.183	0.304
Weight gain (g)	78.2^a^	62.9^a,c,d^	41.7^b^	74.1^a,d^	51.4^b,c^	15.6	**0.001**	0.220	**0.001**
TGC	0.061^a^	0.049^a,b^	0.033^b^	0.057^a^	0.040^a,b^	0.010	**0.007**	0.147	**0.002**
Feed intake (g WM/fish)	134	124.71	107.82	120.82	96.28	25.36	0.442	0.747	0.234
FCR	1.70^a^	1.98^a,b^	2.56^b^	1.63^a^	1.88^a^	0.40	**0.006**	**0.033**	**0.002**
VSI	7.63^a^	6.39^a,b^	6.53^a,b^	6.76^a,b^	6.34^b^	0.63	**0.039**	0.584	0.206
HSI	0.89	0.85	0.69	0.76	0.70	0.14	0.132	0.422	0.068
LSI	2.05	1.57	1.60	1.65	1.53	0.34	0.365	0.534	0.534
Survival (%)	93	97	97	97	94	3.4	0.957	0.248	0.275

*Note:* Different lowercase letters show significant differences between dietary groups (*p* < 0.05). Bold numbers indicate a significant effect (*p* < 0.05), where *n* = 3 per diet (except *n* = 6 for VSI, HSI, and LSI). Using ANOVA or Kruskall–Wallis test with diet alone or protein + lipid.

Abbreviations: FCR, feed conversion ratio; FI, feed intake; HSI, hepatosomatic index; LSI, liposomatic index; SE, pooled standard error; SGR, specific growth rate; TGC, thermal growth coefficient; VSI, viscerosomatic index; WG, weight gain.

**Table 3:** Carcass composition, apparent digestibility coefficient (ADC), nutrient intake, nutrient retained, and nutrient retention efficiency (mean ± SE) of lake whitefish fed diets Bluewater commercial control (BCC), low-protein–high-lipid (LPHL), low protein low lipid (LPLL), high-protein–high-lipid (HPHL), or high-protein–low-lipid (HPLL) for 16 weeks.

Carcass composition (%)	BCC	LPHL	LPLL	HPHL	HPLL	SE	*p*-Values		
							Diet	Protein	Lipid
Dry matter	21.5	20.7	20.9	21.2	20.8	0.1	0.278	0.247	0.876
Crude protein	17.0	16.7	17.5	17.6	17.4	0.1	0.095	0.154	0.228
Crude lipid	11.3	9.4	9.0	10.2	9.2	0.3	0.080	0.787	0.385
Gross energy (MJ/kg)	8.4	7.7	7.8	8.0	7.7	0.1	0.446	0.441	0.881
Apparent digestibility coefficient (ADC; %)									
Dry matter ADC	67.2^a^	64.7^b^	61.4^c^	67.3^a^	60.9^c^	1.1	**0.001**	0.996	**0.008**
Protein ADC	80.7	81.7	81.8	81.1	80.1	0.4	0.711	0.270	0.734
Lipid ADC	99.2^a^	99.0^a^	98.9^ab^	99.4^a^	97.4^b^	0.2	**0.006**	0.768*⁣*^*∗*^	0.280*⁣*^*∗*^
Gross energy ADC	75.6	74.4	69.7	74.7	72.1	1.1	0.052	0.765	0.062
Nutrient intake (g/fish)									
Crude protein intake	68.0	50.3	55.0	67.4	44.8	4.5	0.230	0.998	0.220
Crude lipid intake	14.6	22.0	9.7	17.6	14.7	1.9	0.224	0.499	0.239
Gross energy intake (MJ/kg/fish)	26.0	19.7	21.7	26.2	14.8	2.0	0.221	0.788	0.208
Nutrient retained (g/fish)									
Protein retained	11.5^a^	8.7^b^	8.0^b^	14.1^a^	9.4^c^	0.7	**0.001**	**0.030** *⁣* ^*∗*^	**0.009** *⁣* ^*∗*^
Lipid retained	11.1^a^	3.1^b^	1.1^c^	7.2^b^	4.6^b^	1.2	**0.003**	0.215	**0.022**
Gross energy retained (MJ/kg/fish)	7.0^a^	3.6^b^	2.6^b^	5.8^a^	4.6^a^	0.5	**0.008**	0.322	**0.048**
Nutrient retention efficiency (%)									
Protein retention efficiency	17.3	19.1	14.8	21.6	22.9	1.5	0.484	0.114	0.692
Lipid retention efficiency	79.5^a^	23.3^b^	10.2^b^	43.3^b^	59.5^a^	7.8	**0.012**	0.416	0.482
Gross energy retention efficiency	27.3	19.5	12.7	22.8	30.4	2.4	0.217	0.158	0.388

*Note:* Different lowercase letters show significant differences between dietary groups (*p* < 0.05). Bold numbers indicate significance (*p* < 0.05), where *n* = 3. Asterix (*⁣*^*∗*^) represents a significant interaction effect. Using ANOVA or Kruskall–Wallis test with diet alone, protein + lipid or protein × lipid.

Abbreviations: ADC, apparent digestibility coefficient; SE, pooled standard error.

**Table 4:** Alpha-diversity indices (mean ± SE) of diet and intestinal bacteria from lake whitefish fed diets Bluewater commercial control (BCC), low-protein–high-lipid (LPHL), low-protein–low-lipid (LPLL), high-protein–high-lipid (HPHL), or high-protein–low-lipid (HPLL) for 16 weeks and before the start of the experimental period (Day 0).

Alpha diversity	Day 0	BCC	LPHL	LPLL	HPHL	HPLL	Feed	SE	*p*-Values		
									Diet	Protein	Lipid
Coverage (%)	95.5^a^	92.5^a^	96.7^a^	75.3^a^	68.9^a^	85.5^a^	61.1^b^	5.7	**0.001**	0.219	0.104
No. of OTUs	16	27	13	37	23	53	91	11	**0.038**	0.319	0.182
Shannon	0.76	0.99	0.59	1.76	1.03	1.91	2.91	0.34	0.119	0.591	0.259
Chao-1	54	80	34	82	61	151	306	41	0.088	0.265	0.144

*Note:* Different lowercase letters show significant differences between dietary groups (*p* < 0.05). Bold number indicate significant effect (*p* < 0.05), where *n* = 3–6 per treatment.

Abbreviations: OTU, operational taxonomic unit; SE, pooled standard error.

## Data Availability

Datasets are available upon reasonable request to the corresponding author.
